# Prevalence of osteoporosis in patients with diabetes mellitus: a systematic review and meta-analysis of observational studies

**DOI:** 10.1186/s12902-022-01260-8

**Published:** 2023-01-03

**Authors:** Xueying Liu, Fuhua Chen, Lei Liu, Qiu Zhang

**Affiliations:** Department of Endocrinology, First Affiliated Hospital of Anhui Medical University, Hefei, 230032 China

**Keywords:** Osteoporosis, Diabetes mellitus, Systematic review, Meta-analysis, Observational studies

## Abstract

**Background:**

Osteoporosis (OP) and diabetes mellitus (DM) are two major healthcare issues in the world. Numerous population based-studies have reported an increased prevalence of OP among individuals with DM, though, estimates vary significantly.

**Purpose:**

The objective of this study is to estimate the prevalence of OP in patients with DM.

**Methods:**

To identify relevant literature, PubMed, Embase, Medline, CBM and Cochrane Library were searched for studies published from inception till July 2022, The search was conducted, and studies were included without countries and language restrictions. For full-text articles included in the study, the references were also independently searched. Random inverse variance-weighted models were used by Stata version 17.0 to estimate the prevalence of OP in patients with diabetes across studies. The heterogeneity was examined with I^2^ via the χ^2^ test on Cochrane’s Q statistic. Subgroup analysis and meta-regression were used to explore potential sources of heterogeneity. Egger’s test was used to assess publication bias.

**Results:**

A high OP prevalence of 27.67% (95% confidence interval (CI) 21.37-33.98%) was found in a pooled analysis of 21 studies involving 11,603 T2DM patients. Methodological value of the included articles was high, with only three medium-quality studies and no low-quality studies. A significantly high heterogeneity (I^2^ = 98.5%) was observed.

**Conclusions:**

Worldwide, a high prevalence of OP was found in patients with T2DM. Therefore, strong measures to prevent and treat osteoporosis in diabetic patients are required.

**Trial registration:**

This study has been registered on PROSPERO, number CRD42021286580.

**Supplementary Information:**

The online version contains supplementary material available at 10.1186/s12902-022-01260-8.

## Introduction

Osteoporosis (OP), one of the most common metabolic skeletal disorders, is characterized by decreased bone mass and increased destruction of bone microstructure, which consequently increases bone fragility and fracture risk [1]. OP tends to occur in older people and individuals with predisposing health condition [2] and has been considered as a serious public health concern attributable to its high morbidity, mortality, and healthcare costs. Some risk factors for OP have been identified, some of which are complex owing to the multiple mechanisms involved, such as diabetes mellitus (DM).

DM has developed into a global health concern that poses a major hazard to human health. And it is associated with an increased risk of fracture, particularly the hip fracture, despite normal or high bone mineral density (BMD). Nowadays, the incidences and prevalence of DM are rising sharply around the world. According to the International Diabetes Federation (IDF), the global prevalence of DM was estimated at 9.3% in 2019, and is expected to increase to 10.2% in 2030 and 10.9% in 2045 [3] Thus emphasizing the need to pay more attention and consideration to this disease.

In the past few years, multiple research has focused on the relationship between type 2 diabetes mellitus (T2DM) and OP [4, 5]. In China, a meta-analysis reported that the prevalence of OP in type 2 diabetics was 44.8 and 37.0% in women and men respectively [6] and a cross-sectional study demonstrated that the prevalence of OP was 5.0 and 20.6% among men and women, respectively in individuals aged over 40 years [7]. Estimates vary significantly.

The prevalence of OP has been assessed in postmenopausal women or elderly men with T2DM in a few regions and countries, However, studies on the prevalence of OP in diabetes patients worldwide have not been well documented. We conducted a systematic review and meta-analysis of the prevalence of OP in patients with DM worldwide. The purpose of this study was to provide clinical guidance for prevention, diagnosis, and control strategies in light of the persistently rising prevalence of OP and DM.

## Methods

### Searching strategy and selection criteria

The study was conducted in accordance with the systematic review and meta-analysis of observational studies. The prevalence of OP in patients with T1DM and T2DM was estimated. The study has been registered on the International Prospective Register of Systematic Reviews (PROSPERO; https://www.crd.york.ac.uk/PROSPERO/), with registration number CRD 42021286580.

To identify primary studies on the prevalence of OP in patients with DM, two investigators did a comprehensive search of PubMed, Embase, Medline, CBM and Cochrane Library databases. The studies published from database inception to July 2022 without any country and language barrier. Medical subject headings (MESH), keywords, and free words were used in the retrieval strategy including “osteoporosis”, “bone losses”, “post-traumatic osteoporosis”, “senile osteoporosis”, “age-related osteoporosis”, “involutional osteoporosis”, “diabetes mellitus”, “diabetes”, “hyperglycemia”, “observational study”, “cross-sectional studies”, and “cohort studies”. To evaluate further studies, a manual search was done on the reference list of all selected. The retrieval strategy in PubMed is applicable to other databases, as shown in details in Additional file 1 Appendix a.

Observational studies: cross-sectional and cohort studies related to OP prevalence in patients with DM were fetched. OP is defined as follows: a bone mineral density (BMD) T-score < = − 2.5 SD in one or more of the following regions: lumbar spine, femoral neck, or total hip, or T-score < = − 2.5 SD plus a history of fracture. Case-control studies were excluded because they were unable to provide any information about the incidence or prevalence of disease as no measurements are made in a population-based sample. Case series with a small sample size (less than 50 participants), reviews, conference abstracts, and articles without primary data or explicit description of methods, or both, were also excluded. For studies published in more than one report (duplicate), the most comprehensive study with the largest sample capacity and influence was considered. In research abstracts without full text available, relevant data was included in the abstract, an attempt was made to email the first author or corresponding author to obtain the full text for information extraction and quality assessment of the specification. Articles without a response from the author were discarded. For final inclusion, two investigators independently examined the titles and abstracts of studies retrieved through the literature search. The full texts of possibly eligible publications were collected. All duplicate articles were removed during the screening process.

Two investigators, X. Y Liu and F. H Chen screened all potential studies, and verified key data, including all data on OP prevalence in patients with DM. Disagreements were resolved through discussions until a consensus was achieved.

### Data extraction

Two investigators, X. Y Liu and F. H Chen, independently retrieved relevant data from separate investigations using a planned and standardized data extraction form. Data information was extracted including the first author’s name, year of publication, country, study design, sample size, OP prevalence in diabetes, measuring site, diagnostic criteria for OP, and contained participants (type of diabetes, age, gender, BMI, and sources of participants). Discrepancies were worked out through discussions until a consensus was reached.

### Data processing

The criteria established by Hoy and his teammates in 2012, which consists of ten items, were used to assess the methodological quality of the observational studies [8]. Each item was given a score of 1 (yes) or 0 (no), and the values were added together to obtain an overall quality score that ranged from 0 to 10. The study was divided into three grades based on its total quality scores, with a low (> 8), moderate (6-8), and high (≤ 5) risk of bias respectively [9]. X. Y Liu and F. H Chen independently assessed the methodological quality of the included studies, and disagreements were resolved through discussion.

To summarize the prevalence data, data analysis was conducted by Stata version 17.0 software. In order to minimize the influence of studies with extreme prevalence on the overall estimate, the proportion was first stabilized through the Freeman-Tukey Transformed method before pooling proportion using the random-effect model meta-analysis [9–11]. Heterogeneity was assessed by Cochran’s Q, I^2^, and H statistics [9]. The percentage of differences across studies to total variation was described by I^2^, where an I^2^ value greater than 50% suggests the presence of between-studies heterogeneity [10]. In this study, the pooled OP prevalence in patients with DM was evaluated using a random-effect model with a forest plot with a 95% confidence interval (95%CI). Subgroup analyses were performed to identify the potential heterogeneity between included studies based on age (<= 60 years vs. > 60 years), gender (proportion of female <= 60% vs. > 60%), BMI (obesity vs. non-obesity), study quality (high vs. moderate), published country (China vs. non-China), and DM duration (<= 10 years vs. > 10 years). BMI was calculated as weight in kilograms divided by height in meters squared. Obesity was defined as BMI ≥ 28 kg/m^2^ in China and ≥ 30 kg/m^2^ in other countries [12, 13]. Of 21 studies, two were excluded from the analysis because they did not mention BMI [14, 15].

Further, a meta-regression was conducted to test the association of OP prevalence in diabetics based on age, gender (proportion of female), sample size (number of study participants), and published year. These all factors may potentially explain differences in OP prevalence. At last, Egger’s test was used to assess publication bias, with *P* < 0.10 suggesting significant publication bias [16].

## Results

**Fig. 1:**
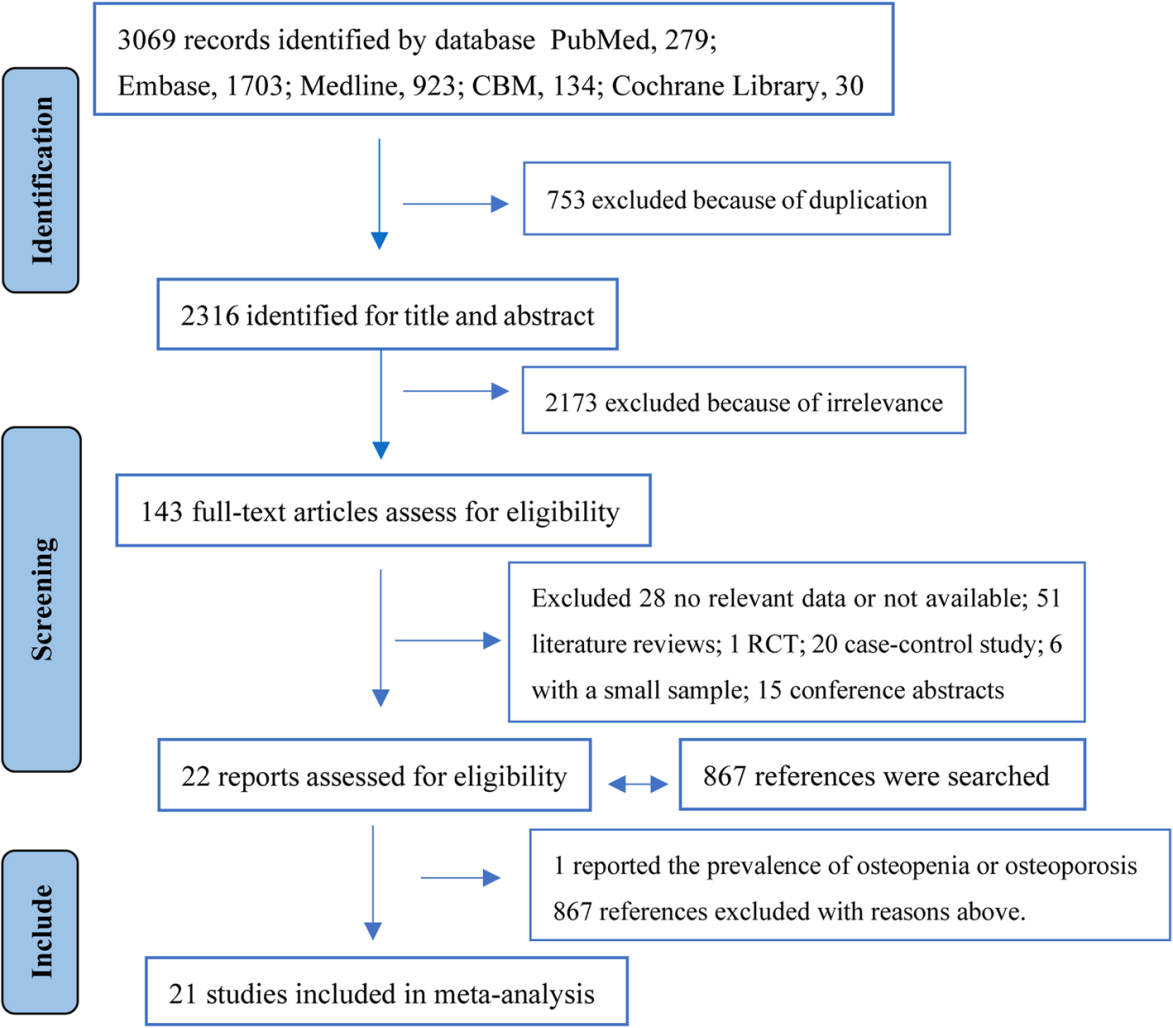
Flowchart of study selection process

The original search began with 3069 studies, of which 753 were excluded due to redundancy. After preliminary screening of titles and abstracts, 2173 non-relevant articles were excluded. The remaining 143 full-text articles were assessed for eligibility; however, a large fraction was excluded for various reasons. Excluded articles comprised 28 articles with no relevant data or not available, 51 literature reviews, 1 randomized controlled trial (RCT), 20 case-control studies, 6 with a small sample size (less than 50 participants), and 15 conference abstracts. The remaining 22 reports were assessed for eligibility, of which one literature reported the prevalence of osteopenia or osteoporosis. From these reports, 867 references were independently searched; however, all articles were excluded because of no relevant data, reviews, not available, and duplicates. Finally, 21 studies including 11,603 individuals with DM, were considered in the meta-analysis [14, 15, 17–34]. The study selection process has been presented in Fig. 1.

Table 1 show the characteristics and OP prevalence in DM patients, for all studies the included studies. Of these only one study included both type 1 and type 2 diabetes [35], and the rest of 21 studies focused on type 2 diabetes and were included in the standardized analysis. The prevalence of OP in patients with DM varied from 7.29 to 53.71% across studies. The methodological quality of included studies was high, with only three medium-quality [17, 20, 24] and no low-quality studies (Table 2).

**Table 1:** General information and features of the included 22 studies

Study	Country	Study design	Skeletal site	DC for OP	*N*	*n*	Percent (%)	Participants and sources
Kanazawa,2019 [26]	Japan	cross-sectional study	The thoracic and LS	T score	309	166	53.70	T2DM, 196 male, 113 female; age 65.3 years; BMI 25.2 kg/m2, from hospital;
Maíra Viégas,2011 [20]	Maceió	cross-sectional study	LS and FN	T score	148	45	30.40	T2DM, postmenopausal women, age 61.87 ± 7.85 (41–80 years); from outpatient clinic, Hospital
Abdulameer,2018 [25]	Malaysia	cross-sectional study	The heel bone	T score and QUS	450	100	22.20	T2DM, 231(51.3%)male, 219(48.7%)female; age 62.67 ± 9.24 years; BMI 26.36 ± 4.39 kg/m2, from the outpatient diabetes clinic, hospital;
Maryam Ghodsi,2021 [31]	Iran	cross-sectional study	LS (L2–L4), hip, and FN	T score or Z score	165	23	13.92	T2DM, 63 male,102 female; age, total 50.86 (11.17), male 51.02 (12.85), female 50.76(10.07); BMI 29.40 (4.32) kg/m2, from community.
AL-Homood,2017 [23]	Arabia	cross-sectional study	LS (L2–L4) and FN	T score	170	50	29.41	T2DM, females patients; age 56.3 ± 8.5 years; BMI 32.2 ± 6.2 kg/m2; from hospital.
Yan Guo,2020 [27]	China	cross-sectional study	The heel bone	T score	1073	434	40.45	T2DM, male 382, female 691(64.4%); age 69.09 ± 6.53; BMI 25.29 ± 3.31; from communities.
Afshinnia, 2007 [18]	USA	cross-sectional study	LS (L2–L4), TH	T score	159	54	33.96	T2DM, 26 male, 133 female; age 72.3 ± 10.4 years; BMI 28.5 ± 5.4 kg/m2; from hospital
Yaturu, 2009 [19]	USA	cross-sectional study	LS and FN	T score	550	97	17.63	T2DM, 550 male,age 67.5 ± 0.38 (50 to 76) years; BMI 30.08 ± 0.2; from hospital
Schwartz, 2005 [14]	USA	cohort study	FN and TH	T score	480	66	13.75	T2DM, 222 (46.25%) female; age 70-90 years; from university
Roma’n, 2004 [17]	Spain	cross-sectional study	The calcaneal	T score	92	20	21.70	T2DM, 56 females and 36 males; age 63.3 ± 9.1 years; BMI 29.6 ± 6.1 kg/m2; from a tertiary care hospital.
Lingna Fang, 2021 [30]	China	cross-sectional study	LS and hip	T score	187	58	31.02	T2DM, 82 male, 105 female; age male 65.23 ± 9.34, female 65.08 ± 8.28 years; BMI male 24.9 ± 3.99, female 25.61 ± 3.62 kg/m2; from hospital.
Jianbo Li, 2014 [22]	China	cross-sectional study	LS (L1–L4) and FN	T or Z score	258	133	51.55	T2DM, postmenopausal women; age non-OP 59.6 (48.7, 65.3), OP 63.3 (50.2, 67.7); BMI non-OP 23.7 (2.2), OP 22.4 (2.7); from hospital.
Karimifar, 2012 [21]	Iran	cross sectional study	FN and LS (L2-L4)	T score	200	78	39	T2DM; postmenopausal women; age 66.91 ± 5.78 years; BMI 31.65 ± 4.42 kg/m2; from hospital.
Bruckner, 2014 [35]	Germany	cross-sectional study	Self-reported	T score	398	T1, 14 T2, 43	T1, 9.03 T2, 17.7	155 T1DM, 243 T2DM; T1DM, 139 (71 male, 68 female), T2DM, 243 (115 male, 128 female) age T2DM male, 62.7 ± 8.5 female, 62.9 ± 8.5 BMI T1D male 25.2 ± 3.3, female 24.6 ± 2.9, T2D male 28.9 ± 4.5,female 29.7 ± 5.3 kg/m2; from hospital.
Yufeng Li, 2020 [28]	USA	cross-sectional study	LS (L2-4)	QCT	629	106	16.85	T2DM, male 342, female 287(46%); age 55.12 ± 9.75 years; BMI 27.55 ± 3.87 kg/m2; from communities.
Junyan Li, 2021 [32]	China	cross-sectional study	LS (L1–L4) and left hip	T score	164	65	39.63	T2DM, postmenopausal women; age 45-84 years, NBG 55.17 ± 6.59, OAG 62.17 ± 7.71, OPG 65.78 ± 8.1 years; from hospital.
Min Qiu, 2020 [29]	China	cross sectional study	LS (L1–L4) and FN	T score	147	26	17.69	T2DM, 84 male, 63 female; age NBG 53.4 ± 3.2, OAG 55.7 ± 4.1 OPG 57.6 ± 5.0 years; BMI NBG 25.3 ± 2.1,OAG 25.0 ± 1.7, OPG 24.9 ± 1.7 kg/m2; from hospital.
Shuangling Xiu, 2019 [5]	China	cross-sectional study	LS (2–4), FN, and the hip	T score	370	121	32.70	T2DM, male 176, female 194; age 67.6 ± 5.78 years; BMI 25.81 ± 3.66; from hospital.
Xiaojuan Xu,2021 [34]	China	cross-sectional study	LS (L1–L4), FN, and the hip	T score	933	220	23.58	T2DM, 535 male> = 50 years and 398 postmenopausal women); age male 63.0 (59.0, 67.0), female 64.0 (59.0, 70.0); BMI male 24.58 ± 3.29, female 24.58 (22.03, 27.30); from hospital.
Yang Wu, 2021 [33]	China	cross-sectional study	LV, FN, TH	T score	631	374	59.27^A^	T2DM, male; age 57.3 ± 12.0 years; BMI 24.9 ± 3.6 kg/m2; from hospital.
L. Zhou, 2017 [24]	China	cross-sectional study	LS (L1–L4), proximal femur	T score	99	27	27.30	T2DM, postmenopausal women; age 62 ± 8 years; BMI 27.6 ± 4.2 kg/m2; from hospital.
Xueyu Li, 2021 [15]	China	cross-sectional study	Existing database	T score	4777	348	7.30	T2DM, male 52% (2482); age 64.2 ± 14.2 years, from hospital

Study: the first author‘s name or first name, published year

Continuous variables are expressed as means ± standard deviation or medians with interquartile range

Categorical variables are expressed as numbers with percentages

T1, type 1 diabetes mellitus; T2, type 2 diabetes mellitus; LS, lumbar spine; FN, femoral neck; LV, lumbar vertebra, FN, femoral neck; TH, total hip;

QUS, Quantitative Ultrasound Scan

NBG, normal BMD group; OAG, osteopenia group; OPG, osteoporosis group;

OP, osteoporosis; N, sample size of diabetic patients; n, the number of OP in diabetes; DC, diagnostic criteria; BMI, body mass index

A, the prevalence of osteopenia or osteoporosis

**Table 2:** Quality assessment of the 22 included studies

Study	Q1	Q2	Q3	Q4	Q5	Q6	Q7	Q8	Q9	Q10	TS	QG
Kanazawa,2019	1	1	1	1	1	1	1	1	1	1	10	high
Maíra Viégas,2011	0	0	1	1	1	1	1	1	1	1	8	moderate
Abdulameer,2018	1	1	1	1	1	1	1	1	1	1	10	high
Maryam Ghodsi,2021	0	1	1	1	1	1	1	1	1	1	9	high
AL-Homood,2017	0	1	1	1	1	1	1	1	1	1	9	high
Yan Guo,2020	1	1	1	1	1	1	1	1	1	1	10	high
Afshinnia MD, 2017	0	1	1	1	1	1	1	1	1	1	9	high
Yaturu,2009	0	1	1	1	1	1	1	1	1	1	9	high
Schwartz,2004	1	1	1	1	1	1	1	1	1	1	10	high
Roma’n,2004	0	0	1	1	1	1	1	1	1	1	8	moderate
Lingna Fang,2021	0	1	1	1	1	1	1	1	1	1	9	high
Jianbo Li,2014	0	1	1	1	1	1	1	1	1	1	9	high
Karimifar,2012	0	1	1	1	1	1	1	1	1	1	9	high
Bruckner,2014	1	1	1	1	1	1	1	1	1	1	10	high
Yufeng Li,2020	1	1	1	1	1	1	1	1	1	1	10	high
Junyan Li,2021	0	1	1	1	1	1	1	1	1	1	9	high
Min Qiu,2020	0	1	1	1	1	1	1	1	1	1	9	high
Shuangling Xiu,2019	1	1	1	1	0	1	1	1	1	1	10	high
Xiaojuan Xu,2021	1	1	1	1	1	1	1	1	1	1	10	high
Yang Wu, 2021	0	1	1	1	1	1	1	1	1	1	9	high
L. Zhou, 2017	0	0	1	1	1	1	1	1	1	1	8	moderate
Xueyu Li, 2020	1	1	1	1	1	1	1	1	1	1	10	high

Study: first author, Published year; TS, total scores; QG, quality grade;

External validity: Q1-Q4; Internal validity: Q5-Q10

Q1: Was the study’s target population a close representation of the national population in relation to relevant variables?

Q2: Was the sampling frame a true or close representation of the target population?

Q3: Was some form of random selection used to select the sample, or was a census undertaken?

Q4: Was the likelihood of nonresponse bias minimal?

Q5: Were data collected directly from the subjects (as opposed to a proxy)?

Q6: Was an acceptable case definition used in the study?

Q7: Was the study instrument that measured the parameter of interest shown to have validity and reliability?

Q8: Was the same mode of data collection used for all subjects?

Q9: Was the length of the shortest prevalence period for the parameter of interest appropriate?

Q10: Were the numerator(s) and denominator(s) for the parameter of interest appropriate?

**Fig. 2:**
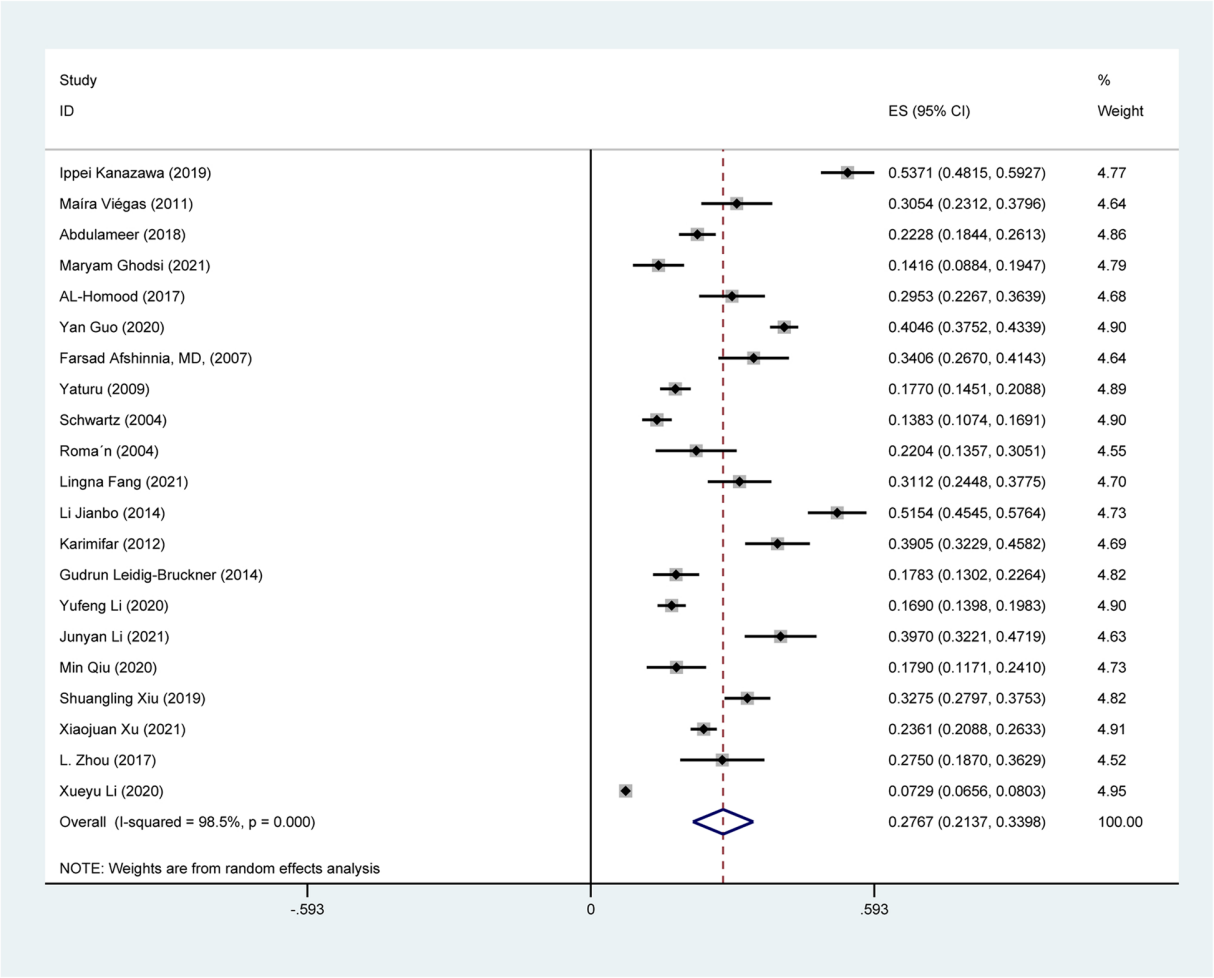
The forest plot of pooled OP prevalence in patients with DM

A pooled prevalence of 27.67% (95% CI 21.37-33.98%) of OP was found in patients with DM (Fig. 2). The heterogeneity tests indicated significant differences between individual studies (I^2^ = 98.5%, *P* < 0.001). To explore the potential source of heterogeneity, several subgroups were defined and a meta-regression was carried out.

**Fig. 3:**
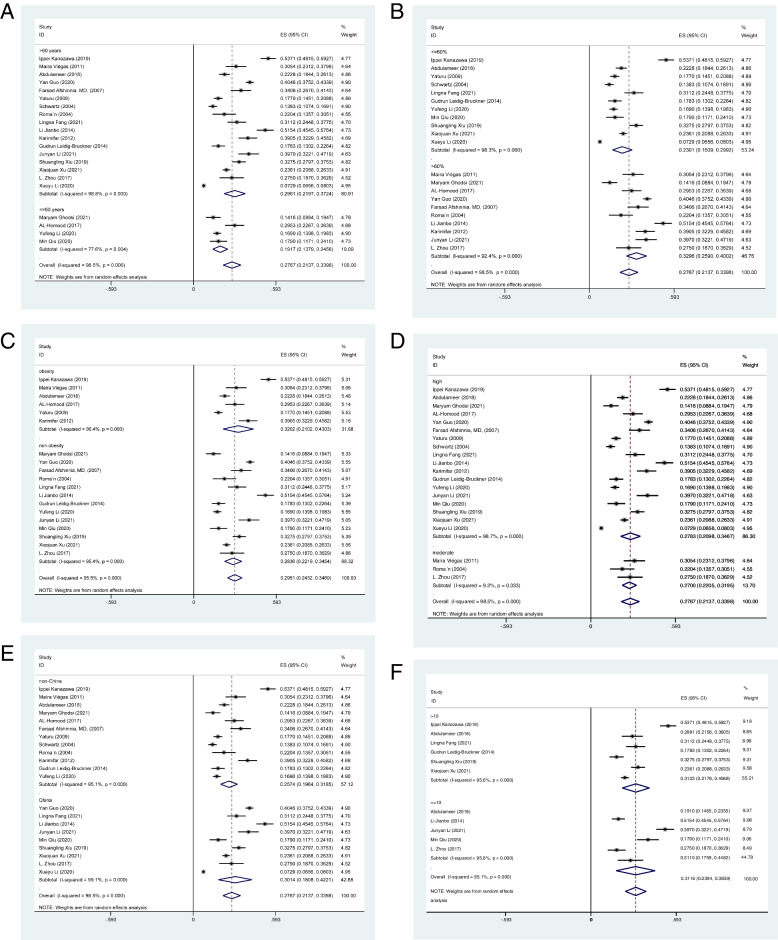
**A.** The forest plot of OP prevalence in patients with DM based on age. **B.** The forest plot of OP prevalence in patients with DM based on gender. **C.** The forest plot of OP prevalence in patients with DM based on BMI. **D.** The forest plot of OP prevalence in patients with DM based on study quality. **E.** The forest plot of OP prevalence in patients with DM based on country. **F.** The forest plot of OP prevalence in patients with DM based on DM duration

**Fig. 4:**
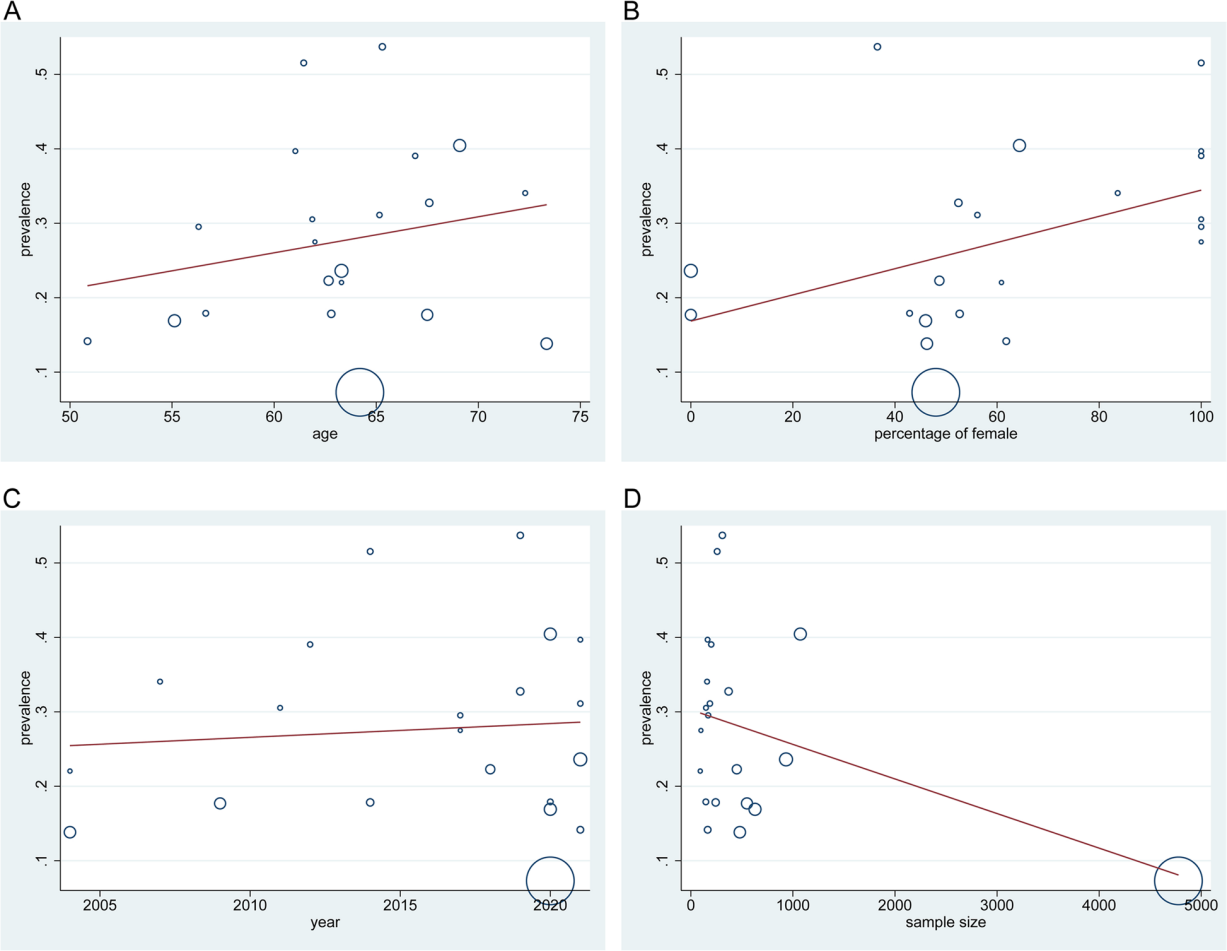
**a.** Meta-regression: age at OP testing and OP prevalence in T2DM. y-Axis: % of T2DM study participants with OP. x-Axis: age in years when tested for OP (*P* = 0.354). **b.** Meta-regression: gender at OP testing and OP prevalence in T2DM. y-Axis: % of T2DM study participants with OP. x-Axis: % of female when tested for OP (*P* = 0.050). **c.** Meta-regression: published year and OP prevalence in T2DM. y-Axis: % of OP with T2DM. x-Axis: published year (*P* = 0.715). **d.** Meta-regression: sample size and OP prevalence in T2DM. y-Axis: sample size of studies. x-Axis: sample size (*P* = 0.086)

Age. The prevalence of OP in diabetes patients aged 60 or younger was lower (19.17% [95% CI 13.79-24.56%]), compared to the older individuals (29.61% [95% CI 21.97-37.24%]) (Fig. 3A). Fig. 4a shows that there was no significant relationship between age at testing for OP prevalence in diabetes patients (*P* = 0.354).

Gender. The prevalence of OP was higher in studies with a higher proportion of female participants (32.96% [95% CI 25.90-40.02%]) than in that with a lower proportion (23.01% [95% CI 16.09-29.92%]) (Fig. 3B). The meta-regression suggested a positive association between the OP prevalence and female proportion; however, the results were not statistically significant (*P* = 0.050, Fig. 4b).

BMI. The OP prevalence in obese patients with DM was higher (32.02% [95% CI 21.02-43.03%]) than that in non-obese (28.36% [95% CI 22.19-34.54%]) (Fig. 3C).

Quality of studies. The studies of high quality embrace slightly higher prevalence (27.83% [95% CI 20.98-34.67%]), compared to studies with moderate quality (27.00% [95% CI 22.05-31.95%]) (Fig. 3D).

Country. The prevalence of OP in diabetics in China (30.14% [95% CI 18.08-42.21%]) was higher than that in non-China (25.74% [95% CI 19.54-31.85%]) (Fig. 3E).

DM duration. There was a slightly higher OP prevalence in those who suffered diabetes for more than 10 years (31.23% [95% CI 21.78-40.68%]), compared with those less than 10 years (31.10% [95% CI 17.59-44.62%]) (Fig. 3F).

Other possible factors. The meta-regression indicated that there was no association between the prevalence of OP in patients with DM and the publishing year (*P* = 0.715; Fig. 4c), as well as the sample size (*P* = 0.086; Fig. 4d). Nevertheless, the results were not statistically significant.

**Fig. 5:**
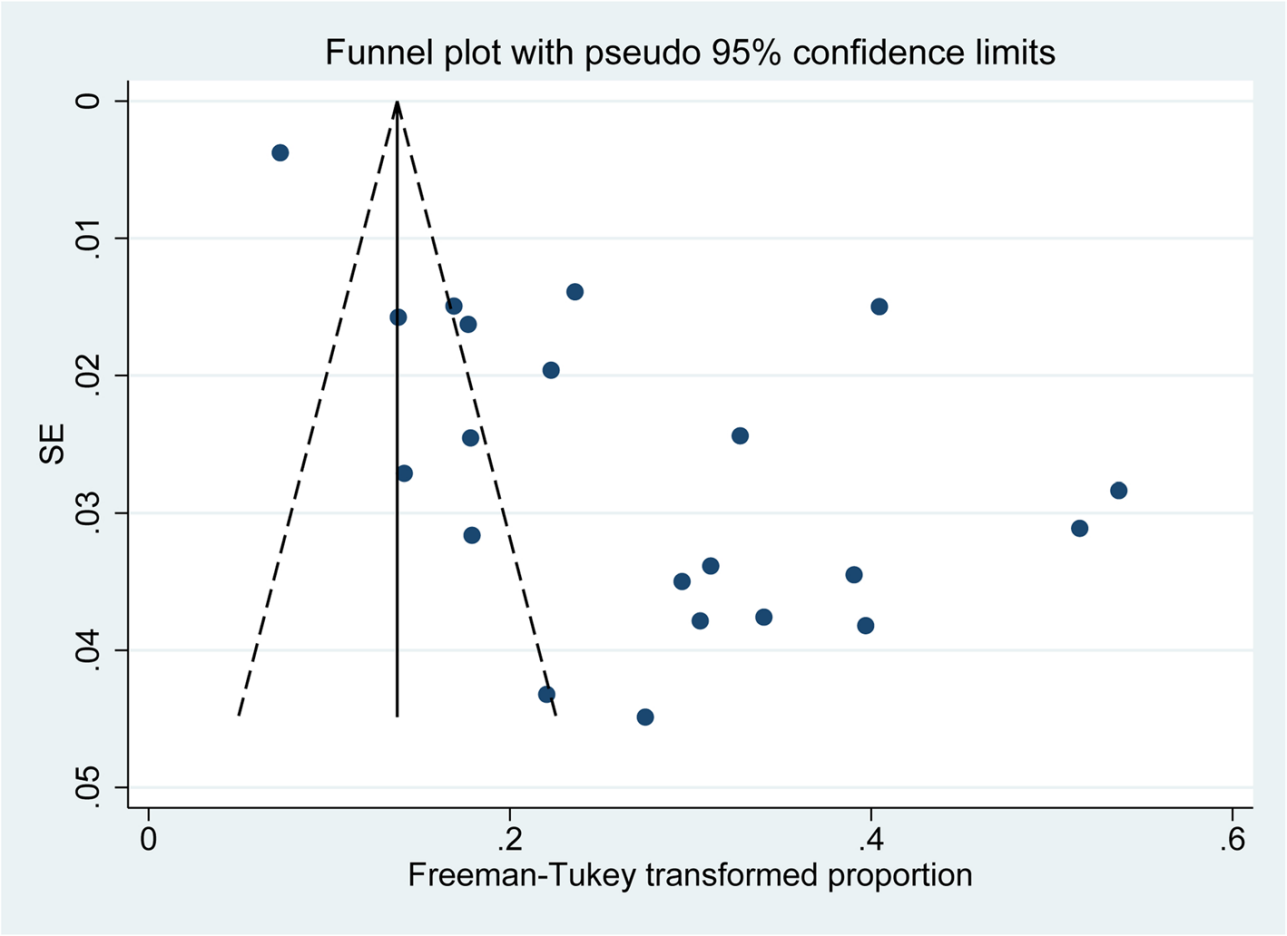
Funnel plot of included studies

Additionally, a funnel plot was made, which indicated the existence of publication bias where larger studies seem more likely to be published if they show high OP prevalence for patients with DM (Fig. 5).

## Discussion

Based on the 21 analyzed articles, 11,603 individuals with DM, a OP prevalence of 27.67% (95% CI 21.37-33.98%) was observed in this meta-analysis. The current study provides direction for future studies on bone health in diabetics with many studies recommending population-based bone mineral density (BMD) studies in patients with DM.

Only a small number of studies published estimated the prevalence of OP in patients with T2DM worldwide, and a wide range reflects high inconsistency of the prevalence evaluation. This systematic review and meta-analysis found that the prevalence of OP in diabetics varies between 7.29 and 53.71%. The comparison groups in the different studies are not uniform and the primary outcomes not consistent, that makes it difficult to compare one another and arrive at valid conclusions.

In this study, although some minor differences in OP prevalence were identified in subgroups, the prevalence of OP in diabetics could not be influenced significantly by age, gender (female proportion), BMI, country, published year, and sample size. This is notable given that the OP is more prevalent in certain populations, such as obese patients [36], postmenopausal women or older men [37, 38]. For instance, data from National Health and Nutrition Examination Survey (NHANES) from 2005 to 2010 highlighted that 16.2% of adults aged 65 and over had OP at the lumbar spine or femur neck. The age-adjusted prevalence of osteoporosis at either skeletal site was higher among women (24.8%) compared to men (5.6%). In the United States, the unadjusted prevalence was higher among adults aged 80 and over (25.7%) than for adults aged 65 to 79 (12.8%) [39]. Age and gender differences could lead to obvious distinction in the prevalence of OP.

Although we also found that the OP prevalence in diabetics was higher in postmenopausal women, the elderly, obesity group, the effect was not significant. Several confounders could account for this contradiction. Bone mineral density (BMD) is one of the factors that must not be ignored in studies on OP. For the majority of individuals with OP, BMD T-score < − 2.5 SD or less was chosen as the diagnostic criteria produced by the World Health Organization (WHO) (Table 1). The diagnostic criteria provided a tool that could be used in epidemiological studies to quantify the prevalence of OP and confirmed the importance of low BMD in the pathogenesis of fragility fractures. Whereas, the utility of BMD as a clinical indicator of OP is limited, as BMD is only one of a lot of important risk factors for fracture, and the majority of vulnerability fractures occur in groups with BMD values above this threshold [40]. These suggested that BMD could not be a faultless clinical indicator of OP and osteoporotic fractures. Subsequently, BMD varies by skeletal site. In our included studies, Viegas M and colleagues [20] reported the prevalence of OP was 30.4% at lumbar spine (LS) and 9.5% at femoral neck (FN). Furthermore, based on NHANFS (2005-2014) data, significant trends (quadratic or linear) were observed for the femur neck (mean T-score and OP in both sexes; low bone mass in women) but not for the lumbar spine. The trend in femur neck status was somewhat U-shaped, with prevalence being most consistently significantly higher (by 1.1-6.6 percentage points) in 2013-2014 than in 2007-2008. FN trend was unchanged even after adjusting for variations in BMI, smoking, milk intake, and physician’s diagnosis of OP between surveys. In 2013-2014, the percent of older adults with OP was 6% at the femur neck, 8% at the lumbar spine, and 11% at either site [41].

The current study showed that the prevalence of OP in individuals with T2DM cannot be significantly affected by BMI. The aforementioned studies revealed the complex relation between BMI and BMD. Al-Homood et al. [23] showed that BMI protects against a decrease in BMD, such a finding has been claimed by Chen et al. in their study among elderly type 2 diabetic men [42]. Nonetheless, Doğan, A and his teammates [43] confirmed BMD values increased as BMI values increased and the effect of a high BMI on femoral neck and L2-L4 BMD among older men and women, but the effect of age was not shown above 75 years of age. In addition, glycemic control, use of oral agents, individual’s lifestyles, the method used for measurements and the study design were not considered in this study.

Research quality can part explain the significant heterogeneity. In sub-analyses, there was no difference between moderate-quality studies (*P* = 0.333). Another one explanation for the significant variation in reported OP prevalence may be publication bias. The small and inconsistent sample size in selected studies was one limitation in drawing stable conclusions with regard to OP occurrence in patients with DM, further prospective and high quality studies are warranted on a larger scale, which includes more potentially confounding factors.

## Conclusions

This systematic review and meta-analysis revealed that OP is a common comorbidity in diabetics. We found a 27.67% prevalence of OP in patients with DM worldwide. This finding highlights the case for action to implement the control of OP in diabetic patients. Such efforts include the improvement of access to laboratory testing, training of professionals for OP management, and facilitation of access to comprehensive therapy for OP and T2DM.

## Supplementary Information

**Additional file 1.** The detailed retrieval strategy in PubMed, Embase, Cochrane Library, Medline and CBM databases.

## Data Availability

All data generated or analyzed during this study are included in this published article and its supplementary information files.
