# Supplementary material for: Prevalence of osteoporosis in patients with diabetes mellitus: a systematic review and meta-analysis of observational studies
Source: BMC Endocr Disord. 2023 Jan 3;23:1. doi: 10.1186/s12902-022-01260-8 (PMC9809067; doi:10.1186/s12902-022-01260-8)
Supplement: Supplementary file 1 — Additional file 1. The detailed retrieval strategy in PubMed, Embase, Cochrane Library, Medline and CBM databases. [file 12902_2022_1260_MOESM1_ESM.pdf]

PubMed(until July 2022)

No. Query results

#1 "Osteoporosis"[Mesh] **60737**

#2((((((((((((((((((((Osteoporoses[Title/Abstract]) OR (Osteoporosis, Post-Traumatic[Title/Abstract])) OR (Osteoporosis, Post Traumatic[Title/Abstract])) OR (Post-Traumatic Osteoporoses[Title/Abstract])) OR (Post-Traumatic Osteoporosis[Title/Abstract])) OR (Post-Traumatic Osteoporosis[Title/Abstract])) OR (Osteoporoses, Senile[Title/Abstract])) OR (Senile Osteoporoses[Title/Abstract])) OR (Osteoporosis, Involutional[Title/Abstract])) OR (Senile Osteoporosis[Title/Abstract])) OR (Osteoporosis, Age-Related[Title/Abstract])) OR (Osteoporosis, Age Related[Title/Abstract])) OR (Bone Loss, Age-Related[Title/Abstract])) OR (Age-Related Bone Loss[Title/Abstract])) OR (Age-Related Bone Losses[Title/Abstract])) OR (Bone Loss, Age Related[Title/Abstract])) OR (Bone Losses, Age-Related[Title/Abstract])) OR (Age-Related Osteoporosis[Title/Abstract])) OR (Age Related Osteoporosis[Title/Abstract])) OR (Age-Related Osteoporoses[Title/Abstract])) OR (Osteoporoses, Age-Related[Title/Abstract])

3735

#3 ("Osteoporosis"[Mesh]) OR (((((((((((((((((((((Osteoporoses[Title/Abstract]) OR (Osteoporosis, Post-Traumatic[Title/Abstract])) OR (Osteoporosis, Post Traumatic[Title/Abstract])) OR (Post-Traumatic Osteoporoses[Title/Abstract])) OR (Post-Traumatic Osteoporosis[Title/Abstract])) OR (Post-Traumatic Osteoporosis[Title/Abstract])) OR (Osteoporoses, Senile[Title/Abstract])) OR (Senile Osteoporoses[Title/Abstract])) OR (Osteoporosis, Involutional[Title/Abstract])) OR (Senile Osteoporosis[Title/Abstract])) OR (Osteoporosis, Age-Related[Title/Abstract])) OR (Osteoporosis, Age Related[Title/Abstract])) OR (Bone Loss, Age-Related[Title/Abstract])) OR (Age-Related Bone Loss[Title/Abstract])) OR (Age-Related Bone Losses[Title/Abstract])) OR (Bone Loss, Age Related[Title/Abstract])) OR (Bone Losses, Age-Related[Title/Abstract])) OR (Age-Related Osteoporosis[Title/Abstract])) OR (Age Related Osteoporosis[Title/Abstract])) OR (Age-Related Osteoporoses[Title/Abstract])) OR (Osteoporoses, Age-Related[Title/Abstract]))

62266

#4 "Diabetes Mellitus"[Mesh] **483126**

#5 (diabetes[Title/Abstract]) OR (hyperglycemia[Title/Abstract]) **636784**

#6 ("Diabetes Mellitus"[Mesh]) OR ((diabetes[Title/Abstract]) OR (hyperglycemia[Title/Abstract]))

761022

#7 (((("observational study"[Title/Abstract] OR "Cross Sectional Studies"[Title/Abstract])) OR (cohort studies[Title/Abstract])) OR (((("observational studies"[Title/Abstract] OR "Cross Sectional Study"[Title/Abstract])) OR (cohort study[Title/Abstract])))) **639618**

#8 ((("Osteoporosis"[Mesh]) OR (((((((((((((((((((((Osteoporoses[Title/Abstract]) OR (Osteoporosis, Post-Traumatic[Title/Abstract])) OR (Osteoporosis, Post Traumatic[Title/Abstract])) OR (Post-Traumatic Osteoporoses[Title/Abstract])) OR (Post-Traumatic Osteoporosis[Title/Abstract])) OR (Post-Traumatic Osteoporosis[Title/Abstract])) OR (Osteoporoses, Senile[Title/Abstract])) OR (Senile Osteoporoses[Title/Abstract])) OR (Osteoporosis, Involutional[Title/Abstract])) OR (Senile Osteoporosis[Title/Abstract])) OR (Osteoporosis, Age-Related[Title/Abstract])) OR (Osteoporosis, Age Related[Title/Abstract])) OR (Bone Loss, Age-Related[Title/Abstract])) OR (Age-Related Bone Loss[Title/Abstract])) OR (Age-Related Bone Losses[Title/Abstract])) OR

(Bone Loss, Age Related[Title/Abstract])) OR (Bone Losses, Age-Related[Title/Abstract])) OR (Age-Related Osteoporosis[Title/Abstract])) OR (Age Related Osteoporosis[Title/Abstract])) OR (Age-Related Osteoporoses[Title/Abstract])) OR (Osteoporoses, Age-Related[Title/Abstract]))) AND (("Diabetes Mellitus"[Mesh]) OR ((diabetes[Title/Abstract]) OR (hyperglycemia[Title/Abstract])))) AND ((((("observational study"[Title/Abstract] OR "Cross Sectional Studies"[Title/Abstract])) OR (cohort studies[Title/Abstract])) OR (((("observational studies"[Title/Abstract] OR "Cross Sectional Study"[Title/Abstract])) OR (cohort study[Title/Abstract])))) **279**

Embase(until July 2022)

No. Query Results

#1 'osteoporosis'/exp **146540**

#2 osteoporoses:ti,ab,kw OR 'osteoporosis, post-traumatic':ti,ab,kw OR 'post-traumatic osteoporoses':ti,ab,kw OR 'osteoporosis, senile':ti,ab,kw OR 'senile osteoporoses':ti,ab,kw OR 'age-related bone loss':ti,ab,kw OR ' osteoporosis, Involutional':ti,ab,kw **1180**

#3 #1 OR #2 **147062**

#4 'diabetes mellitus'/exp **1161432**

#5 diabetes:ti,ab,kw OR hyperglycemia:ti,ab,kw **984056**

#6 #4 OR #5 **1340884**

#7 'observational study':ab,ti OR 'cross-sectional study':ab,ti OR 'cohort analysis':ab,ti OR 'case control study':ab,ti OR 'cohort study':ab,ti OR 'cohort studies':ab,ti OR 'observational studies':ab,ti OR 'cross-sectional studies':ab,ti **1039329**

#8 #3 AND #6 AND #7 **1703**

Cochrane Library (until July 2022)

ID Search Hits

#1 MeSH descriptor: [Osteoporosis] explode all trees **4350**

#2 (osteoporoses):ti,ab,kw OR (Osteoporosis, Post Traumatic):ti,ab,kw OR (post-traumatic osteoporoses):ti,ab,kw OR (post-traumatic osteoporosis):ti,ab,kw OR (Osteoporoses, Senile):ti,ab,kw **21**

#3 (Senile Osteoporoses):ti,ab,kw OR (Osteoporosis, Involutional):ti,ab,kw OR (Senile Osteoporosis):ti,ab,kw OR (Osteoporosis, Age-Related):ti,ab,kw OR (steoporosis, Age Related):ti,ab,kw **287**

#4 (Bone Loss, Age-Related):ti,ab,kw OR (Age-Related Bone Loss):ti,ab,kw OR (Age-Related Bone Losses):ti,ab,kw OR (Bone Losses, Age-Related):ti,ab,kw OR (Age-Related Osteoporosis):ti,ab,kw **209**

#5 (Age Related Osteoporosis):ti,ab,kw OR (Age-Related Osteoporoses):ti,ab,kw OR (Osteoporoses, Age-Related):ti,ab,kw **691**

#6 #1 or #2 or #3 or #4 or #5 **4975**

#7 MeSH descriptor: [Diabetes Mellitus] explode all trees **35342**

#8 (diabetes):ti,ab,kw OR (diabetes mellitus type 1):ti,ab,kw OR (iabetes mellitus type 2):ti,ab,kw OR (hyperglycemia):ti,ab,kw **95213**

#9 #7 or #8 **97860**

#10 (observational study):ti,ab,kw OR (observational studies):ti,ab,kw OR (cross sectional study):ti,ab,kw OR (cross sectional studies):ti,ab,kw OR (cross-sectional study):ti,ab,kw **36732**

#11 (cross-sectional studies):ti,ab,kw OR (cohort study):ti,ab,kw OR (cohort studies):ti,ab,kw OR (case-control study):ti,ab,kw OR (case-control studies):ti,ab,kw **75893**

#12 #10 or #11 **99252**

#13 #6 and #9 and #12 **30**

Medline(until July 2022)

No. Query Results

#1 MH=Osteoporosis **48632**

#2 TS=(osteoporosis OR Osteoporosis, Post-Traumatic OR Osteoporosis, Post Traumatic OR post-traumatic osteoporoses OR post-traumatic osteoporosis OR Osteoporoses, Senile OR Senile Osteoporoses OR Osteoporosis, Involutional OR Senile Osteoporosis OR Osteoporosis, Aged Related OR Osteoporoses, Senile OR Senile Osteoporoses OR Osteoporosis, Involutional OR Senile Osteoporosis OR Osteoporosis, Aged Related OR Age-Related Osteoporosis OR Age Related Osteoporosis OR Aged Related Osteoporoses) **96560**

#3 (#1) OR #2 **96560**

#4 MH=Diabetes Mellitus **131579**

#5 TS=(diabetes OR diabetes mellitus type 1 OR diabetes mellitus type 2 OR hyperglycemia) **732756**

#6 (#4) OR #5 **732756**

#7 AB=(observational study OR observational studies OR cross sectional study OR cross sectional studies OR cross-sectional study OR cross-sectional studies OR cohort study OR cohort studies) **1058522**

#8 ((#3) AND #6) AND #7 **923**

CBM (until July 2022)

No. Query Results

#1 "osteoporosis" [Common field] OR "bone loss " [Common field] OR "senile osteoporosis" [Common field] OR "postmenopausal osteoporosis "[Common field] OR "Age-related bone loss" [Common field] **60827**

#2 "diabetes" [Common field] OR "hyperglycemia" [Common field] OR "Type 1 diabetes" [Common field] OR "Type 2 diabetes" [Common field] **446508**

#3 "observational study" [Common field] OR "cross sectional study" [Common field] OR "cohort study" [Common field] **696861**

#4 #1 AND #2 AND #3 **134**
